# IMA Genome - F13: Draft genome sequences of *Ambrosiella cleistominuta*, *Cercospora brassicicola, C. citrullina, Physcia stellaris,* and *Teratosphaeria pseudoeucalypti*

**DOI:** 10.1186/s43008-020-00039-7

**Published:** 2020-09-24

**Authors:** P. Markus Wilken, Janneke Aylward, Ramesh Chand, Felix Grewe, Frances A. Lane, Shagun Sinha, Claudio Ametrano, Isabel Distefano, Pradeep K. Divakar, Tuan A. Duong, Sabine Huhndorf, Ravindra N. Kharwar, H. Thorsten Lumbsch, Sudhir Navathe, Carlos A. Pérez, Nazaret Ramírez-Berrutti, Rohit Sharma, Yukun Sun, Brenda D. Wingfield, Michael J. Wingfield

**Affiliations:** 1 Department of Biochemistry, Genetics and Microbiology, Forestry and Agricultural Biotechnology Institute (FABI), University of Pretoria, Private Bag x20, Hatfield, Pretoria, 0028 South Africa; 2 Department of Conservation Ecology and Entomology, Stellenbosch University, Private Bag X1, Matieland, 7602 South Africa; 3 Institute of Agricultural Sciences, Banaras Hindu University, Varanasi, 221005 India; 4 Field Museum, Department of Science and Education, Grainger Bioinformatics Center, Chicago, IL USA; 5 Center of Advanced Study in Botany, Institute of Science, Banaras Hindu University, Varanasi, 221005 India; 6 Departamento de Farmacología, Farmacognosia y Botánica, Facultad de Farmacia, Universidad Complutense de Madrid, Plaza de Ramón y Cajal s/n, 28040 Madrid, Spain; 7 Agharkar Research Institute, G.G. Agharkar Road, Pune, 411004 India; 8 Department of Plant Protection, EEMAC, Facultad de Agronomía, UdelaR, Paysandú, Uruguay; 9 National Centre for Microbial Resource, National Centre for Cell Science, S.P, Pune University, Pune, 411 007 India

**Keywords:** Ambrosia beetle, *Cercospora*, *Brassica rapa subsp. rapa*, Foliose lichens, *Lagenaria siceraria*, *Physcia*, *Teratosphaeria*, Eucalyptus leaf pathogen

## Abstract

Draft genomes of the fungal species *Ambrosiella cleistominuta*, *Cercospora brassicicola, C. citrullina, Physcia stellaris,* and *Teratosphaeria pseudoeucalypti* are presented. *Physcia stellaris* is an important lichen forming fungus and *Ambrosiella cleistominuta* is an ambrosia beetle symbiont. *Cercospora brassicicola* and *C. citrullina* are agriculturally relevant plant pathogens that cause leaf-spots in brassicaceous vegetables and cucurbits respectively. *Teratosphaeria pseudoeucalypti* causes severe leaf blight and defoliation of *Eucalyptus* trees. These genomes provide a valuable resource for understanding the molecular processes in these economically important fungi.

## IMA GENOME-F 13A

### Draft nuclear genome assembly for *Ambrosiella cleistominuta*, an ambrosia beetle symbiont

#### Introduction

Fungi in the genus *Ambrosiella* (*Microascales*, *Ceratocystidaceae*) form an obligate, mutualistic symbiosis with ambrosia beetles from the tribe *Xyleborini* (Mayers et al. 2015). The fungi are dispersed by the ambrosia beetles through a specialised organ called the mycangium (Batra 1963). This sac-like structure is used by the beetle to carry the fungi in a budding yeast-like or arthrospore-like phase from tree to tree (Harrington et al. 2014). Once introduced into a new host tree, the fungus colonizes the wood and the galleries created by the beetle, producing special spores or modified hyphal endings that the insects consume as a food source (Batra 1963; Harrington 2005). Currently, ten species of *Ambrosiella* are formally recognized: *A. beaveri*, *A. nakashimae*, *A. hartigii*, *A. batrae*, *A. xylebori*, *A. roeperi*, *A. grosmanniae* (Mayers et al. 2015), *A. catenulata* (Lin et al. 2017), *A. cleistominuta* (Mayers et al. 2017), and *A. remansi* (Mayers et al. 2019).

*Ambrosiella cleistominuta* was described from the mycangium of the ambrosia beetle *Anisandrus maiche* (Mayers et al. 2017). That beetle is native to Asia, but has recently become invasive in the USA, often infecting flood-stressed *Cornus florida* trees in Ohio (Ranger et al. 2015). *Ambrosiella cleistominuta* was the only species constantly isolated from the mycangia of these invasive beetles. Ascomata were subsequently identified in laboratory cultures of the fungus, as well as in artificially infested stem segments of *Cornus florida* (Mayers et al. 2017). This was a surprising finding as ambrosia fungi are generally considered to be strictly asexual (Farrell et al. 2001; Harrington 2005).

Here we report a draft nuclear genome assembly for *Ambrosiella cleistominuta*. The complete genome sequence of *A. xylebori* is already publicly available (Vanderpool et al. 2017), and the addition of this genome opens the door for comparative genomic studies. Genome-based comparative studies have already provided a better understanding of the biology for many *Ceratocystidaceae* species (van der Nest et al. 2015; Simpson et al. 2018; Sayari et al. 2019; van der Nest et al. 2019), and the availability of another genome for this family will strengthen such studies in future.

#### Sequenced strain

**USA**: *Ohio*: Wayne County, near Barnard Rd., isol. from an *Anisandrus maiche* female caught in-flight, 8 Jul. 2015, *C. Ranger* (CBS 141682, BPI 910177 – dried culture).

#### Nucleotide sequence accession number

This Whole Genome Shotgun project for *Ambrosiella cleistominuta* isolate CBS 141682 has been deposited at DDBJ/ENA/GenBank under the accession JABFIG000000000. The version described in this paper is version JABFIG010000000.

#### Materials and methods

*Ambrosiella cleistominuta* isolate CBS 141682 was obtained from the Westerdijk Fungal Biodiversity Institute in Utrecht, The Netherlands (formerly the CBS-KNAW Fungal Biodiversty Centre) and grown on 2% malt extract agar (MEA: 2% w/v, Biolab, South Africa) at 25 °C. A 14 d old culture was used for genomic DNA isolation which was sent to the Central Analytical Facility at the University of Stellenbosch (Stellenbosch, South Africa). The isolated DNA was used to prepare a 400 bp single-read library that was sequenced on the Ion Torrent Ion S5 system (Thermo Fisher Scientific, Johannesburg, South Africa) with the Ion 530 Chip Kit. Additional genomic DNA was isolated from 14 d old cultures grown on a cellophane sheet on 2% MEA using a DNeasy Plant Mini Kit (Qiagen, Germany). This DNA was sent to the Agricultural Research Council Biotechnology Platform (ARC-BTP; Pretoria, South Africa) where it was used to prepare a pair-end library with an insert size of 500 bp. The Illumina HiSeq 2500 instrument (Illumina, San Diego, CA) was used to generate 125 bp length reads from both ends of the insert.

The IonTorrent reads obtained were used for read-error correction and assembly with SPAdes v. 3.14.0 using custom K-values (21, 33, 43, 55, 67, 77, 87, 99, 101, 111, 121, 125), flagging the data as IonTorrent (Bankevich et al. 2012) and applying the “careful” option to reduce mismatches. The generated IonTorrent assembly scaffolds were then used as trusted contigs together with the Illumina data in a second assembly using SPAdes with default settings. The resulting assembly was assessed for completeness using the Benchmarking Universal Single Copy Orthologs tool (BUSCO v. 2.0.1) (Simão et al. 2015) and the Fungi_odb9 (2017-02–13) dataset. An estimation of the number of protein coding genes in the genome were made by the de novo prediction software AUGUSTUS using the *Fusarium graminearum* gene models (Stanke et al. 2006; Keller et al. 2011), while general genome statistics (genome length, GC content, N50, L50 and largest contig size) were calculated using QUAST v. 5.0.1 (Mikheenko et al. 2018).

The *tef1-α* gene region was extracted from the draft genome assembly and, together with previously published *tef1-α* sequences from *A. catenulata* (Lin et al. 2017), *A. beaveri*, *A. nakashimae*, *A. hartigii*, *A. batrae*, *A. xylebori*, *A. roeperi*, *A. grosmanniae* (Mayers et al. 2015), *A. cleistominuta* (Mayers et al. 2017) and *A. remansi* (Mayers et al. 2019), were used for phylogenetic analysis. The dataset consisting of these sequences were used to create a maximum likelihood phylogeny through the Phylogeny.fr online tool (Dereeper et al. 2008, 2010). The “one click” option was used that includes a MUSCLE alignment (Edgar 2004) and Gblocks curation step (Castresana 2000) that precedes phylogenetic analysis using PhyML (Guindon and Gascuel 2003). For branch support, an approximate likelihood ratio test was employed (Anisimova and Gascuel 2006).

#### Results and discussion

**Fig. 1:**
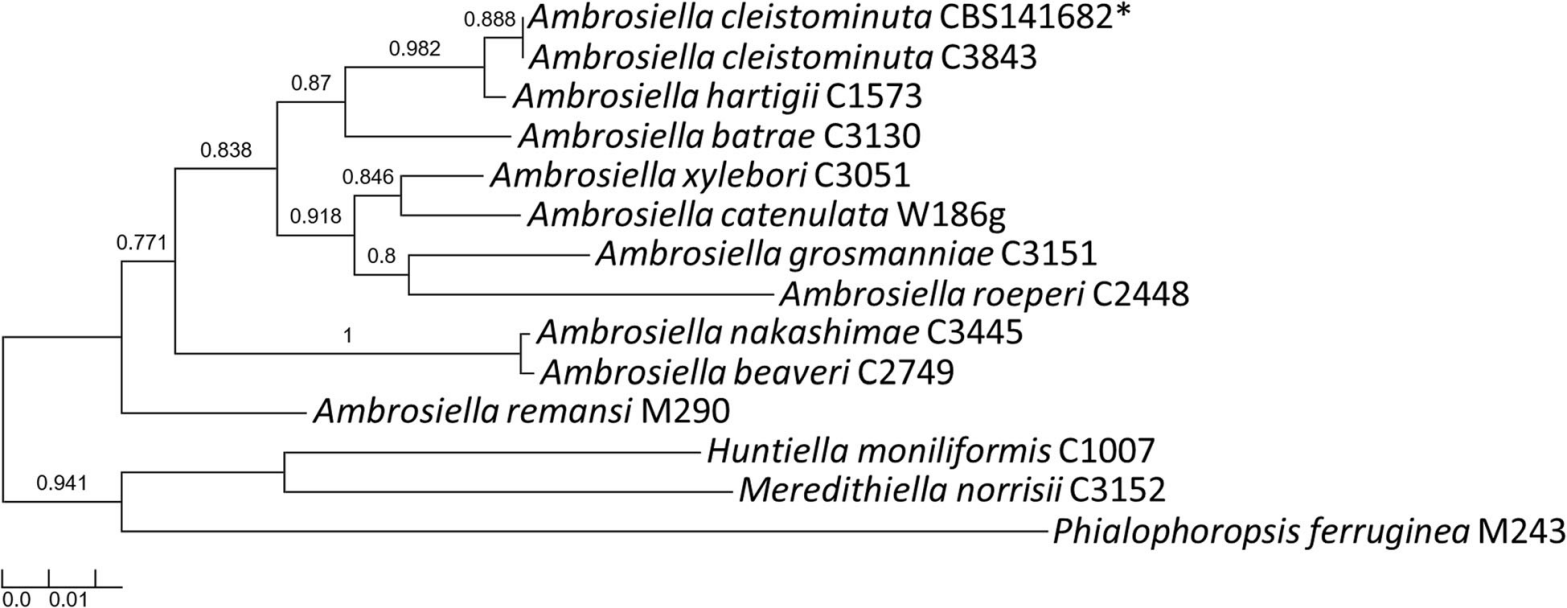
A maximum-likelihood phylogeny showing the position of the *Ambrosiella cleistominuta* isolate (indicated with an asterisk) for which the current genome was generated. Ten known species of *Ambrosiella* are represented, with *Huntiella*, *Meredithiella* and *Phialophoropsis* species used as the outgroup. Approximate likelihood ratio test values for branch support are shown as ratios

The draft genome sequence of *A. cleistominuta* (Fig. 1) presented here has a total length of 27,307,632 bp. This assembly was present in 1517 contigs, with the longest being 555,520 bp and a total of 527 contigs longer than 1000 bp, and had a N50 value of 108,394 bp and a L50 value of 73. The genome had a GC content of 46.71% and an average coverage of 38x, while AUGUSTUS predicted 6611 protein coding genes. BUSCO analysis reported a completeness score of 98.3%. This was based on the analysis of 290 orthologs, with 285 present as complete and five copies completely absent.

The genome assembly of *A. cleistominuta* compared well to that of the previously published *A. xylebori* genome assembly (Vanderpool et al. 2017). The former genome has a higher estimated completeness (98.3% vs 94.1%) and a larger number of predicted genes (6611 vs 6503) compared to *A. xylebori*. However, the draft genome of *A. cleistominuta* is significantly more fragmented than that of *A. xylebori* (1517 vs 59 contigs), and this is further reflected in the larger N50 value of *A. xylebori* (108,394 bp vs 1,073,584 bp). Combining a mate-pair library with the short-insert Illumina dataset during sequencing of the *A. xylebori* genome (Vanderpool et al. 2017) likely lowered fragmentation of the draft assembly, improving contiguity. Mate-pair data has effectively been used in the past to improve the assembly of mammalian (van Heesch et al. 2013), fungal (Wibberg et al. 2015) and plant (Belova et al. 2013) genomes. Similarly, the draft genome for *A. cleistominuta* presented here could be made more contiguous in future by using either mate-pair sequences or long-read sequences. Decreasing the fragmentation for the genome will make it more useful for studies such as whole-genome comparisons.

As *A. cleistominuta* is the first *Ambrosiella* species for which sexual reproduction was documented (Farrell et al. 2001; Harrington 2005; Mayers et al. 2017), the genome assembly reported here will be crucial to understanding the role of the sexual cycle in the biology of this species. Although sexual reproduction has not been observed in the other *Ambrosiella* species, there is growing evidence that asexual species may exhibit cryptic sexuality (Kück and Pöggeler 2009; Dyer and O’Gorman 2012; Ene and Bennett 2014). The genome assembly reported here will not only be useful to studying the mating-type genes of *A. cleistominuta*, but also of other *Ambrosiella* species. Additionally, the availability of a second *Ambrosiella* genome will support efforts to develop molecular tools for population genetic analyses that can be used to evaluate the population level effects of sexual reproduction (Paoletti et al. 2005).

*Authors*: Frances A. Lane*, **Brenda D. Wingfield, and P. Markus Wilken**

*Contact: Frances.Lane@fabi.up.ac.za

## IMA GENOME-F 13B

### Draft genome sequence of *Cercospora brassicicola*, causing White Leaf Spot on *Brassica* species

#### Introduction

**Fig. 2:**
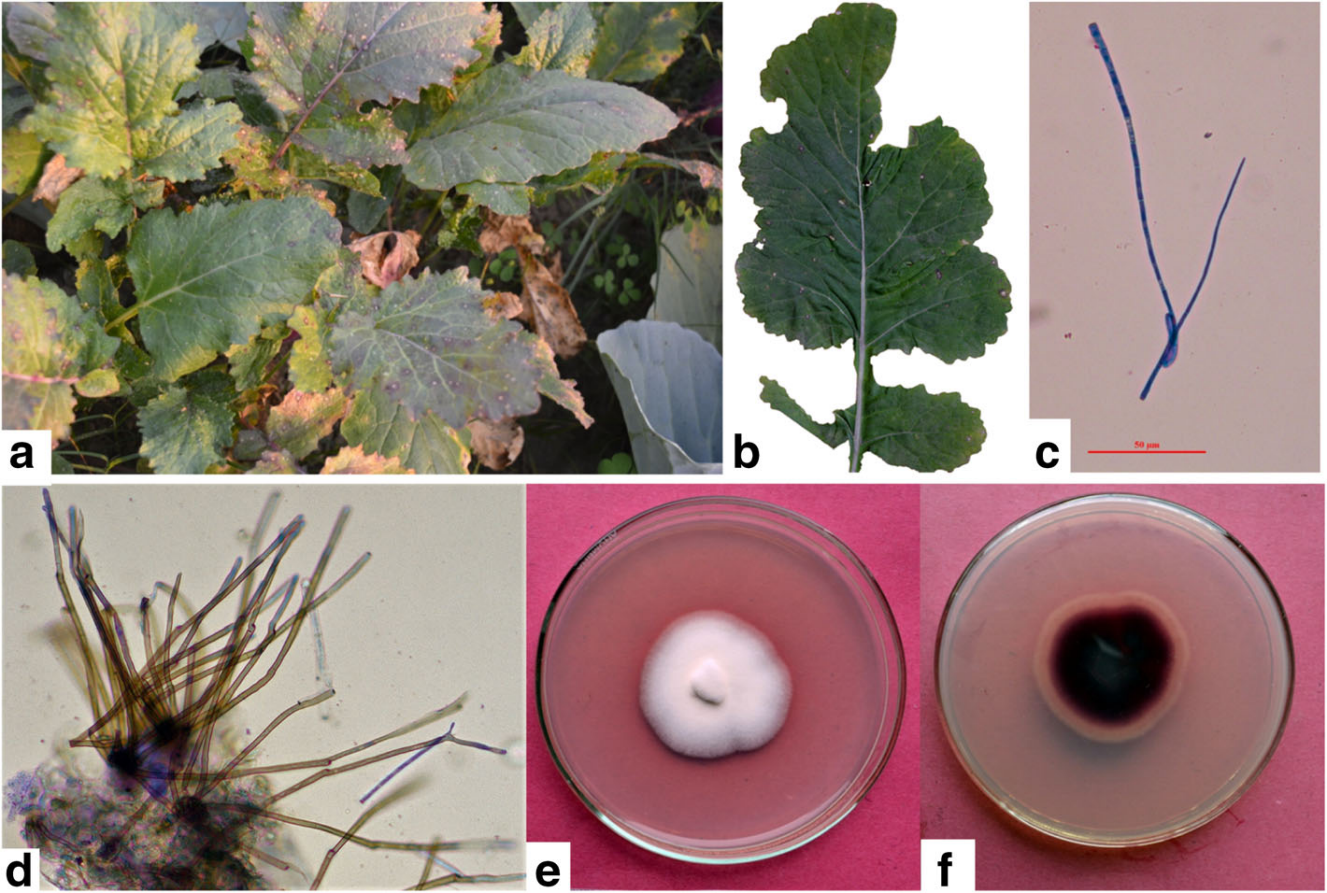
*Cercospora brassicicola* (NFCCI 4678) on *Brassica rapa* subsp. *rapa* **a**-**f**, **a**-**b**. Symptoms on leaf **c**. Conidia **d**. Conidiophores **e**. Pigmented colony on potato dextrose agar (PDA) medium **f**. Inverted pigmented colony. Bars: **c** and **d** = 50 μm

Cercospora white leaf spot on *Brassica* species is caused by *Cercospora brassicicola*. The genus *Cercospora* is one of the largest genera of *Mycosphaerellaceae* in *Capnodiales* (Groenewald et al. 2013). The conidiophores are pale olivaceous to medium brown, rarely branched, multiseptate, 0–7 abruptly geniculate with large spore scar at the sub-truncate tip and are of 3.5–7 × 25–500 μm in size whereas conidia are hyaline, acicular, curved or undulate, indistinctly multiseptate, truncate base, acute tip and of 2–5 × 25–200 μm in size (Fig. 2 C-D) (Chupp 1954). The hosts affected by this pathogen include *Brassica chinensis*, *B. oleracea*, *B. pekinensis*, *B. rapa*, *B. juncea*, *B. nigra*, *B. napobrassicae* (syn. *B. campestris), B. integrifolia*, *B. napus*, and other *Brassica* spp. (Chupp 1954; Hsieh and Goh 1990; Kamal 2010). The disease is widely spread in all tropical and subtropical countries. White leaf spots are circular to angular with a white center, and brown raised border (Fig. 2 A-B) (Chupp 1954). Leaf-spots cause discoloration and decay of petioles and blades, reducing product quality and value. Fields with such infected foliage are either sold on a discount rate or get rejected by the processors (Kahn et al. 2005). According to the U.S. Department of Agriculture standards for mustard and turnip, leaves with more than 10% of the surface area discolored are unsalable (Langston Jr et al. 2005).

Out of 935 species of *Cercospora* reported from India by Kamal (2010), only 1 published genome of *C. canescens* (Chand et al. 2015) exists. During a review of a yet unpublished monograph on the cercosporoid fungi of India, it was found that there is little barcode information available from India. The additional genomic data can aid in better insights into host-pathogen interaction and disease management strategies.

#### Sequenced strain

India: Varanasi, Banaras Hindu University Agricultural Farm, isolated from infected leaf samples of *Brassica rapa subsp. rapa*, Feb. 2018, *R. Chand & S. Sinha* (Cer 68–18; NFCCI 4678).

#### Nucleotide sequence accession numbers

The genome sequence of *Cercospora brassicicola* (NFCCI 4678) has been deposited in the DDBJ/ENA/GenBank databases under the accession number JAASLH000000000; Bio project PRJNA613165; Biosample SAMN14395395. The version described in this paper is version JAASLH010000000. The raw Illumina HiSeq sequence reads are deposited in NCBI-Sequence Read Archives (SRA) under accession SRX7967433. The genome annotation and data on predicted genes have been deposited in Mendeley data with doi number 10.17632/gsdsb9m5w4.1.

#### Materials and methods

##### Isolation and DNA extraction

A monoconidial isolate from infected leaves was grown in 40 ml potato dextrose broth (20% potato dextrose broth w/v) and incubated for 7 d at 25 **±** 1 °C. The fungal material was harvested aseptically after 7 d and genomic DNA was extracted using modified cetyltrimethylammonium bromide (CTAB) extraction protocol (Murray and Thompson 1980). Eppendorf BioPhotometer®D30 was used for the quantification of DNA.

##### PCR amplification and sequencing

Sequencing of the internal transcribed spacer region (ITS), 28S rRNA gene (large subunit-LSU) and 18S rRNA gene (small subunit-SSU) was accomplished by amplification using PCR Thermocycler (Eppendorf MasterCycler 5333) to confirm the identification of the fungal isolate. Primers used for amplification of different regions are as follows: ITS1 and ITS 4 for ITS1–5.8S-ITS2 region (White et al. 1990), LROR and LR7 for LSU region and NS1 and NS5 were used for SSU (https://sites.duke.edu/vilgalyslab/rdna_primers_for_fungi). Amplification was performed using standard PCR conditions. The PCR products were checked on 1.5% agarose gel. The sequencing was outsourced at AgriGenome Labs (Kochi, India).

##### Phylogenetic analysis

**Fig. 3:**
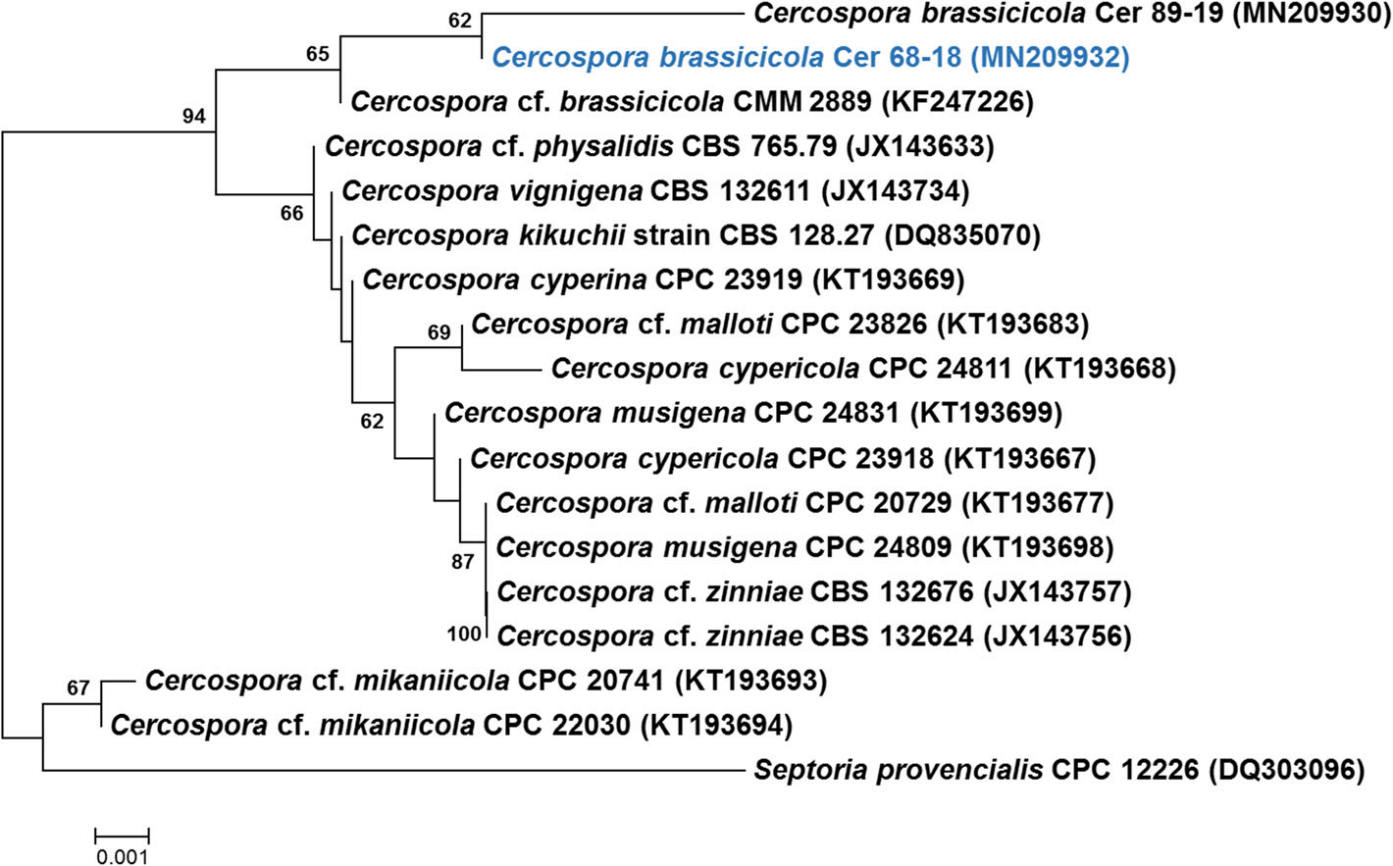
Neighbor-Joining (NJ) tree of the nrITS of *Cercospora brassicicola*. Sequence alignments were produced using Clustal W of Molecular Evolutionary Genetics Analysis (MEGA) software v 7.0 (Kumar et al. 2016). Bootstrap values below 50% were not considered. The tree was rooted to *Septoria provencialis*. The percentage of replicate trees in which the associated taxa clustered together in the bootstrap test (1000 replicates) is shown next to the branches

The seqs thus generated have been submitted at the National Centre for Biotechnology Information (NCBI). The sequences were checked and edited manually using Chromas Pro software (Technelysium). An NCBI BLASTn search for ITS sequence similarity was done with the type database of NCBI (Altschul et al. 1990). Based on the closest similarity of the BLASTn search, the ITS region sequences of ex-type and authentic strains, sequences from Groenewald et al. (2013), and Nguanhom et al. (2015) were retrieved from GenBank. Alignment of sequences was done using Clustal W of Molecular Evolutionary Genetics Analysis (MEGA) software v 7.0 (Kumar et al. 2016). Gaps and missing data were deleted during the sequence alignment. Phylogenetic tree construction using ITS region sequences was done by the Neighbour-Joining method (Fig. 3) (Saitou and Nei 1978). Confidence values for individual branches were determined by bootstrap analyses of 1000 replicates. Bootstrap values below 50% were not considered.

##### Genome assembly and annotation

The library was sequenced with Illumina using a combination of short insert paired-end (2 × 100 bp) and long insert mate-pair (2x250 bp) HiSeq 2500 platform. Initial quality control of the raw sequence reads was performed using FastQC (Andrews 2010). Remnant adapters and low-quality reads with average quality score less than 30 were filtered out in any of the paired end reads using AdapterRemovalV2 v 2.3.1 (Schubert et al. 2016). Finally, unique reads removing duplicated short reads were fetched using FastUniq v 1.1 (Xu et al. 2012). De novo assembly was performed using MaSuRCA v 2.3.2 (Zimin et al. 2013) using the 25, 33, 55, 77, 99, and 127 k-mers. All contigs below 500 bp were discarded. Quality assessment of complete assembly statistics was performed in QUAST v 4.6 (Gurevich et al. 2013). Genome completeness was obtained using BUSCO v 2.0 (Simão et al. 2015). Genes were predicted with AUGUSTUS from the assembled contigs (Stanke and Morgenstern 2005). The predicted gene functions were compared with the UniProt (www.uniprot.org) and NCBI (www.ncbi.nlm.nih.gov) database using the BLASTx v 2.6.0 program with E-value cut-off of 10^− 3^. The best BLASTx hit based on query coverage, identity, similarity score and description of each gene was filtered out. The top BLASTX hit of each gene was studied and the organism name was extracted. The predicted genes were annotated against UniProt for gene ontology in terms of molecular functions, cellular components, and biological Processes.

#### Results and discussion

The assembled draft genome of *C. brassicicola* was estimated to be 38.34 Mb corresponding to 3078 contigs larger than 500 bp with an N50 value of 17,086 bp and an average GC content of 52.84%. Genome completeness analysis (BUSCO) suggests that the assembly covers 96.8% (281/290) (C:281 [S:266, D:157], F:6, M:3, n:290) of the organism’s gene content including 266 complete and single-copy ortholog. AUGUSTUS predicted a total of “11,797” genes in the assembly. The number of predicted genes with significant BLASTx matched with the UniProt database was “10,502” (89.02%). The gene ontology in terms of molecular functions (1027 terms), cellular component (347 terms), and biological process (951 terms) were mapped.

There are very limited whole-genome sequence resources available in the genus *Cercospora*. Those that are available include *C. canescens* (∼34 Mb; Chand et al. 2015), *C. arachidicola* (∼33 Mb; Orner et al. 2015), *C.* cf. *sigesbeckiae* (∼35 Mb; Albu et al. 2017), *C. zeina* (∼37 Mb; Wingfield et al. 2017), *C. sojina* (∼30 Mb; Zeng et al. 2017), *C. beticola* (∼37 Mb; Wingfield et al. 2018a, b), and *C. kikuchii* (∼33 Mb; Sautua et al. 2019).

The genome resource of the *C. brassicicola* reported here is the first from *Brassica rapa subsp. rapa.* This will help in reducing the knowledge gap, which will help in the identification of loci associated with the virulence of the pathogen and in identifying the genetic variants and diagnostic tools.

*Authors*: Ramesh Chand^*^, Shagun Sinha, Sudhir Navathe^*^, **Ravindra N. Kharwar, and Rohit Sharma**

*Contact: sudhir.agro123@gmail.com, rc_vns@yahoo.co.in

## IMA GENOME-F 13C

### Draft genome sequence of *Cercospora citrullina*, causing Leaf Spot on cucurbits

#### Introduction

**Fig. 4:**
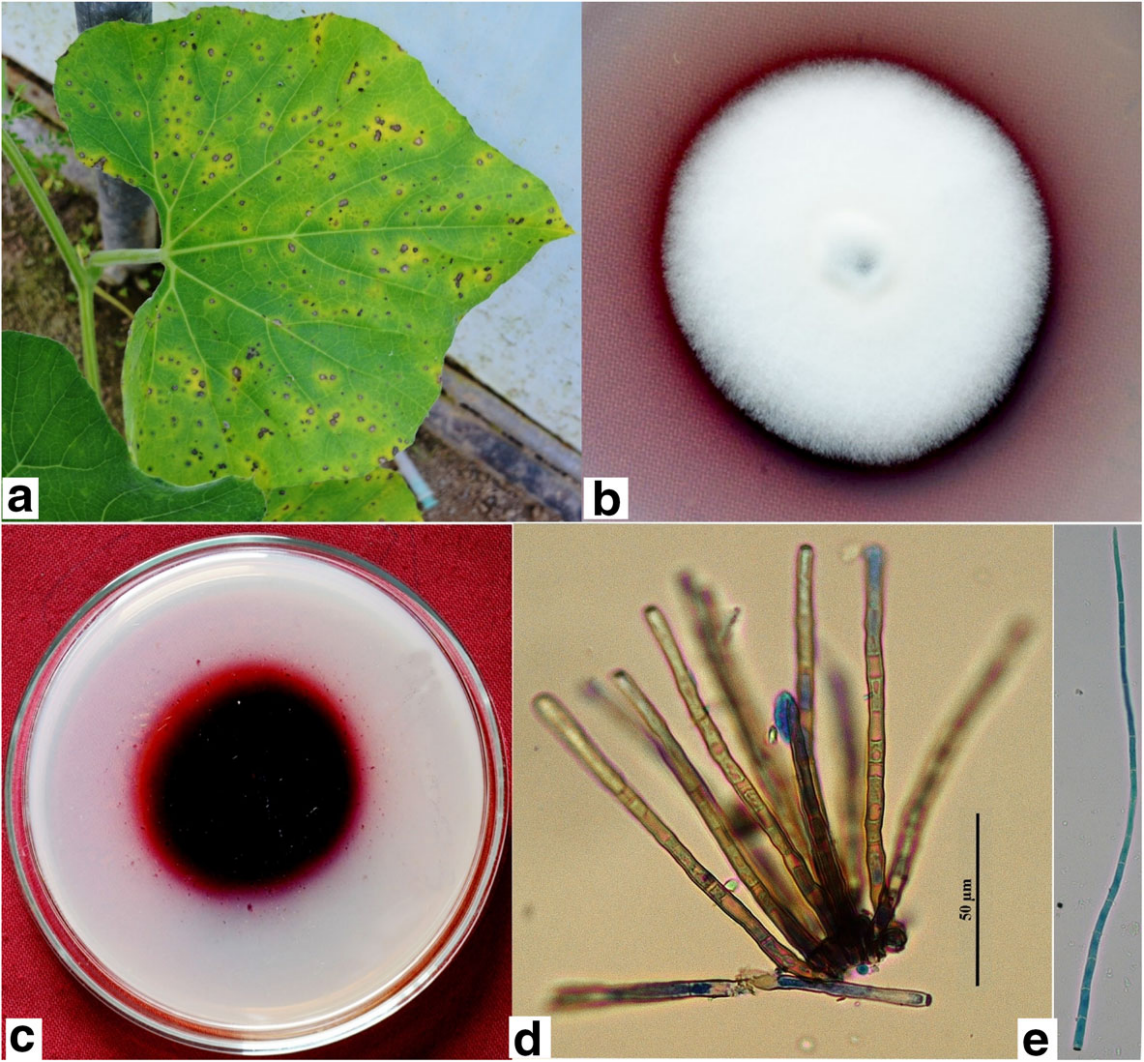
*Cercospora citrullina* (NFCCI 3835) on *Lagenaria siceraria*. **a**. Symptoms on leaf **b**. Colony on potato dextrose agar **c**. Inverted pigmented colony **d**. Conidiophore **e**. Conidia

Cercospora leaf spot on cucurbits is caused by *Cercospora citrullina*. The disease is widespread in tropical and subtropical countries especially in the rainy season when high moisture levels and warm temperatures prevail. Leaf spots on cucurbits are circular to irregular, 0.5–7 mm diam, pale brown or tan to white, sometimes ash-like in appearance usually with a dark brown to black margin surrounded by yellow halo (Fig. 4a) (Chupp 1954). The centre of the spot eventually becomes transparent and brittle. The disease may cause leaf fall (Little 1987). The disease can also reduce fruit size and quality, but economic losses are rarely severe.

Genus *Cercospora* is one of the largest genera of the family *Mycosphaerellaceae* in *Capnodiales* (Groenewald et al. 2013). *Cercospora citrullina* is characterized by presence of conidiophores which are single or in spreading fascicles of 2–30, mostly 2–5, pale to very pale brown, uniform in width or mildly attenuated toward the distal end, occasionally swollen at some point, straight to slightly bent or curved, geniculations varying from none to numerous, multiseptate, not branched, fairly large spore scar at the sub-truncate tip and 4–5.5 × 50–300 μm, rarely 6.5 × 500 μm in size. Conidia are hyaline, acicular, almost never obclavate, straight to strongly curved, indistinctly multiseptate, base truncate, tip acute, 2–4 × 50–220 μm, or even as large as 5.5 × 450 μm in size (Fig. 4d-e) (Chupp 1954). Most of the cucurbitaceous hosts are infected by this pathogen; these include *Benincasa, Bryonia, Citrullus vulgaris*, *Coccinia, Cucumis melo, C. sativus*, *Cucurbita foetidissima*, *C. maxima*, *C. pepo*, *Lagenaria leucantha*, *L. siceraria, Luffa cylindrica*, *Melothria, Momordica charantia*, *M. cochinchinensis*, *M. cordifolia*, *M. foetida*, *Sechium edule*, *Sicana odorifera*, *Telfairia*, *Trichosanthes anguina*, and *T. japonica* (Chupp 1954; Little 1987; Hsieh and Goh 1990; Kamal 2010). The *Cercospora* species on cucurbits invade to such an extent that it is impossible without careful cross inoculations to give host limits and synonymy. There is a lack of morphological differences to separate the morphs of this pathogen on the various hosts (Chupp 1954).

#### Sequenced strain

India: Varanasi, Mathurapur, isolated from infected leaf samples of *Lagenaria siceraria*, Oct. 2009, *R. Chand* (Cerl 37–09; NFCCI 3835).

#### Nucleotide sequence accession numbers

The genome sequence of *Cercospora citrullina* (NFCCI 3835) has been deposited in the DDBJ/ENA/GenBank databases under accession number JAASFF000000000; Bio-project PRJNA613165; Bio-sample SAMN14395396. The version described in this paper is version JAASFF010000000. The raw Illumina HiSeq sequence reads are deposited in the NCBI-Sequence Read Archives (SRA) under accession SRX7980601. The genome annotation and data on predicted genes have been deposited in Mendeley data with doi number 10.17632/hjnwp64z68.1.

#### Materials and methods

##### Isolation and identification

*Cercospora citrullina* (NFCCI 3835) was isolated aseptically on potato dextrose agar medium (PDA) from infected leaves of *Lagenaria siceraria*. The culture was identified and deposited in NFCCI (National Fungal Culture Collection of India, Agharkar Research Institute, Pune, India).

##### DNA extraction and amplification

The monoconidial isolate was grown in 40 ml potato dextrose broth (20% potato dextrose broth w/v) and incubated for 7 d at 25 **±** 1 °C. Fungal material was harvested aseptically after 7 d and genomic DNA was extracted using a modified cetyltrimethylammonium bromide (CTAB) extraction protocol (Murray and Thompson 1980). Eppendorf BioPhotometer®D30 was used for the quantification of DNA. To ascertain authentic identification of the fungal isolate, sequencing of the internal transcribed spacer region (ITS), 28S rRNA gene (large subunit-LSU), and 18S rRNA gene (small subunit- SSU) was accomplished by amplification using PCR thermocycler (Eppendorf 5333 MasterCycler Thermal Cycler). Primers used for amplification of different regions are as follows: ITS1 and ITS 4 for ITS1–5.8S-ITS2 region (White et al. 1990), LROR, and LR7 for LSU region and NS1 and NS5 were used for SSU (https://sites.duke.edu/vilgalyslab/rdna_primers_for_fungi). Amplification was performed using standard PCR conditions. The PCR products were checked on 1.5% agarose gel. The sequencing was outsourced at AgriGenome Labs (Kochi, India).

##### Phylogenetic analysis

**Fig. 5:**
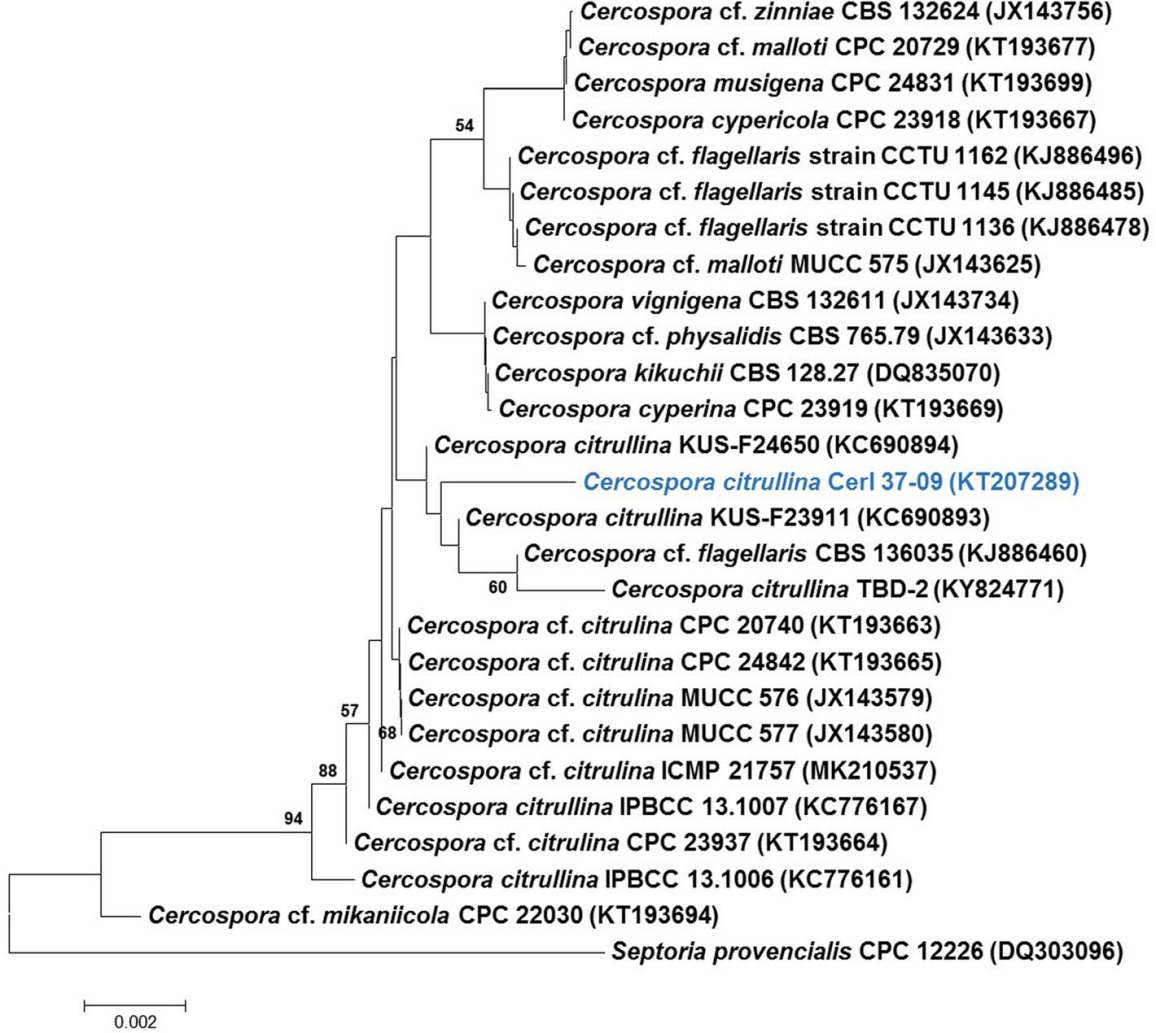
Neighbour-Joining (NJ) tree of the nrITS to identify the sequenced *Cercospora citrullina*. Sequence alignments were produced using MAFFT v 7 (Katoh and Standley 2013) anf further rendered in MEGA7. Bootstrap values below 50% were not considered. The tree was rooted to *Septoria provencialis*. The percentage of replicate trees in which the associated taxa clustered together in the bootstrap test (1000 replicates) is shown next to the branches

The sequences thus generated have been submitted to the National Centre for Biotechnology Information (NCBI). The sequences were checked and edited manually using Chromas Pro software (Technelysium). NCBI BLASTn search for ITS sequence similarity was done with the type database of NCBI (Altschul et al. 1990). Based on the closest similarity of the BLASTn search, the ITS region sequences of ex-type and authentic strains, sequences from Groenewald et al. (2013), and Nguanhom et al. (2015) were retrieved from GenBank. The alignment of sequences was done using MAFFT v 7.0 (Katoh and Standley 2013). The phylogenetic analysis was conducted in MEGA v 7.0 (Kumar et al. 2016). Gaps and missing data were deleted during the sequence alignment. Phylogenetic tree construction using ITS region sequences was done by the Neighbour-Joining method (Fig. 5) (Saitou and Nei 1978). Confidence values for individual branches were determined by bootstrap analyses of 1000 replicates. Bootstrap values below 50% were not considered.

##### Genome sequencing, assembly, and annotation

The library was sequenced on Illumina HiSeq 2500 platform with a combination of short insert paired-end (2 × 100 bp) and long insert mate-pair (2 × 250 bp). Initial quality control of the raw sequence reads was performed using FastQC (Andrews 2010). Remnant adapters and low-quality read with an average quality score of less than 30 in any of the paired-end reads were filtered out using AdapterRemovalV2 v 2.3.1 (Schubert et al. 2016). Finally, unique reads were obtained by removing duplicated short reads using FastUniq v 1.1 (Xu et al. 2012). De novo assembly was performed using Velvet v 1.2.10 (Zerbino and Birney 2008) optimized for k-mer values 31 to 95. All contigs below 500 bp were discarded. Quality assessment of complete assembly statistics was performed in QUAST v 4.6 (Gurevich et al. 2013). Genome completeness was obtained using BUSCO v 2.0 (Simão et al. 2015). Genes were predicted with AUGUSTUS from the assembled contigs (Stanke and Morgenstern 2005). The predicted gene functions were compared with UniProt (www.uniprot.org) and the NCBI database using the BLASTX v 2.6.0 program (www.ncbi.nlm.nih.gov) with an E-value cut-off of 10^− 3^. The best BLASTX hit based on query coverage, identity, similarity score, and description of each gene was filtered out. The top BLASTX hit of each gene was studied and the organism name was extracted. The predicted genes were annotated against UniProt and NCBI database for gene ontology in terms of molecular functions, cellular components, and biological processes.

#### Results and discussion

The genome resource of the *Cercospora citrullina* reported here is the first from *Lagenaria siceraria.* The paired-end and mate-pair sequencing generated approximately 370.23–426.16 Mb raw reads. The assembled draft genome was estimated to be 45.30 Mb corresponding to 358 contigs larger than 500 bp with an N50 value of 637,436 bp, and an average GC content of 52.81%. The largest contig was of 1,464,843 bp. Genome completeness analysis (BUSCO) suggests that the assembly covers 97.9% (284/290) (C: 284 [S: 283, D: 1], F: 3, M: 3, n: 290) of the organism’s gene content that includes 283 complete and single-copy orthologs. AUGUSTUS predicted a total of “9917” genes in the assembly. The number of predicted genes with significant BLASTX matched with the UniProt database was “8856” (89.30%). The gene ontology in terms of molecular functions (1015 terms), cellular component (336 terms), and biological process (958 terms) were mapped. The estimated genome size of *C. citrullina* (∼45 Mb) is comparable to that of other *Cercospora* species including, *C. canescens* (∼34 Mb; Chand et al. 2015), *C. arachidicola* (∼33 Mb; Orner et al. 2015), *C.* cf. *sigesbeckiae* (∼35 Mb; Albu et al. 2017), *C. zeina* (∼37 Mb; Wingfield et al. 2017), *C. sojina* (∼30 Mb; Zeng et al. 2017), *C. beticola* (∼37 Mb; Wingfield et al. 2018a, b) and *C. kikuchii* (∼33 Mb; Sautua et al. 2019). As per Wijayawardene et al. (2017), there are 3000 described species and 700 accepted species of *Cercospora.* However, there are only 18 genome assemblies available until now in global genomic databases (https://www.ncbi.nlm.nih.gov/datasets/genomes/?txid=29002). Therefore, there is a need to fill this knowledge gap with additional genomic data, which can be utilized in the understanding of host-pathogen interaction, loci associated with virulence and disease management strategies. Also, due to the absence of morphological differences in the morphs of *C. citrullina* on the various hosts, it is necessary to have genomic information that can be integrated with morphology to understand host limits and synonymy.

*Authors*: Shagun Sinha, Ramesh Chand^*^, Sudhir Navathe^*^, **Ravindra N. Kharwar, and Rohit Sharma**

*Contact: rc_vns@yahoo.co.in, sudhir.agro123@gmail.com

## IMA GENOME-F 13D

### Draft genome sequence of *Physcia stellaris* (*Physciaceae*)

#### Introduction

*Physcia* is one of 19 genera within the lichen-forming fungal family *Physciaceae* (*Ascomycota*, *Caliciales*) (Lücking et al. 2017). About 80 species of foliose lichens are recognized in *Physcia* (Lücking et al. 2017) and these occur in various habitats, including those rich in nitrogen (Janssen et al. 2007; Boltersdorf and Werner 2014).

In regions with nitrogen pollution, such as cities and regions impacted by agricultural activities, nitrophytic *Physcia* species have been known to outcompete native lichen asemblages (Conti and Cecchetti 2001; Van Herk et al. 2003; Jovan et al. 2012). Therefore, *Physcia* lichens have been used in surveys as bioindicator species. For example, they were used to monitor air health quality in Central Argentina (Estrabou et al. 2011), northeast Italy (Nimis et al. 1991), West Java (Rindita et al. 2015), and South-Eastern Serbia (Stamenković et al. 2010). Moreover, bioindicator lichens can also be used for a more direct estimate of pollution by measuring the pollutants (e.g. metals, metalloids, persistent organic pollutants, radioactive substances, and pesticides) that accumulate in the thallus. These pollutants have potential adverse impacts on human health and the environment. Hence, *Physcia* species have been used to develop lichen elemental bioindicators as part of a national monitoring program for air health quality (Will-Wolf et al. 2017).

We sequenced the first draft genome of the nitrogen-tolerant lichenized fungus *Physcia stellaris.* We collected the specimen for culturing of the mycobiont in Chicago, IL, which is the third largest city of the US and ranked the 16th most air polluted US city in the 2020 “State of the Air” report by the American Lung Association. *Physcia stellaris* is a common species in the midwestern US (Hyerczyk 2005) and characterized by a pale grey thallus with abundant dark brown to black apothecia on the upper surface (Moberg 2002). The species was considered to be cosmopolitan but molecular data show that Australian populations are unrelated and represent a distinct taxon, which is currently accepted as *P. austrostellaris* (Elix et al. 2009). Hence the species delimitation requires further studies, but the species has been confirmed with molecular data to be present in eastern North America and Europe. In addition to the species delimitation efforts of Elix et al. (2009), other previous phylogenetic studies including *Physcia* sequences have been performed to better understand the taxonomy of this group (Lohtander et al. 2000, 2009; Simon et al. 2005; Miadlikowska et al. 2014); however, no full genome in this particular lichen family has peviously been sequenced.

Our presented genome sequence of *P. stellaris* will serve as a reference for future phylogenetic and molecular biodiversity studies of the genus *Physcia,* which will help to determine population sizes, species ranges, and habitats. Understanding *Physcia* habitats and distributions will in turn allow researchers to better utilize the genus as a bioindicator of air polluted regions. As a bioindicator for air pollution, the genome sequence of *P. stellaris* may also help to reveal underlying genomic factors that better adapt these lichens to urban environments with high air pollution compared to other lichens.

#### Sequenced strain

**USA**: *Illinois*: Chicago, 41^o^ 52.00′N, 87^o^ 37.02′W, isolated from thallus on a wooden park bench close to the North entrance of the Field Museum, 7 Aug. 2017, *S.M. Huhndorf* (C03752214F – specimen: #10–5).

#### Nucleotide sequence accession number

The draft whole-genome sequence of the lichenized fungus *Physcia stellaris* (culture collection number #10–5) has been deposited at DDBJ/EMBL/Genbank under the accession number JABSSW000000000. The version described in this paper is version JABSSW010000000.

#### Materials and methods

The *Physcia stellaris* specimen was identified by T.J. Widhelm on 18 Feb. 2020 and is retained in the collections of the Field Museum. Axenic cultures were produced from ascospores and grown on malt-yeast extract agar until the individual cultures reached sufficient sizes for DNA extraction.

High-molecular weight (HMW) DNA extraction of the fungal culture was based on an existing protocol (Hu 2016), with some modifications. About 0.6 g of dried fungal culture material was flash frozen with liquid nitrogen and ground with a ceramic mortar and pestle, then allowed to reach room temperature. The ground material was incubated with 500 μL lysis buffer and 20 μL proteinase K at 64 °C up to 4 h, then cooled on ice for 5 min. To the cool mixture, 100 μL of 5 M KAc was added and incubated for 5 min on ice, then centrifuged at max speed at 4 °C for 10 min. The supernatant was added to 500 μL phenol:chloroform:isoamyl alcohol and centrifuged at max speed at 4 °C for 10 min. The supernatant was added to 500 μL isopropanol and cooled at − 80 °C for 1 h. The isolated HMW DNA was pelleted at max speed at 4 °C for 30 min, washed twice with 1 mL 70% ethanol, and eluted in 50 μL TE buffer.

Isolated HMW DNA was converted into Nanopore libraries with the NBD103 and 1D library kit SQK-LSK 109. The libraries were sequenced on a SpotON R9.4.1 FLO-MIN106 flowcell for 48 h, using a GridIONx5 sequencer. The raw sequencing data was basecalled with Guppy v3.0.3, then adaptor trimmed with Porechops v0.2.3 (https://github.com/rrwick/Porechop). In addition, the same DNA sample was converted into Illumina sequencing libraries with the Hyper Library construction kit from Kapa Biosystems (Roche) and paired-end sequenced for 251 cycles on a MiSeq Illumina sequencer using the MiSeq 600-cycle sequencing kit version 3. All raw Illumina reads were trimmed with Trimmomatic v0.33 (Bolger et al. 2014), setting a quality threshold of 10 (LEADING:10 TRAILING:10). Library construction and sequencing were done at the DNA services facility of the University of Illinois at Urbana-Champaign.

The long-read Nanopore sequences were assembled into continuous contigs with the program Canu v2.0 (Koren et al. 2017) and were then connected by scaffolding with the Nanopore data as a backbone using SSPACE-LongRead v1.1 (Boetzer and Pirovano 2014). These scaffolds were error corrected twice and gap filled with the Nanopore data using Racon v1.4.13 (Vaser et al. 2017) and subsequently polished twice with the trimmed MiSeq Illumina data using Pilon v1.23 (Walker et al. 2014). Genome quality was verified by mapping the long-read Nanopore and short-read Illumina sequences back to the genome with Minimap2 v2.17 (Li 2018) and BWA v0.7.17 (Li and Durbin 2009), respectively. The genome completeness was evaluated with BUSCO v4.0.6 (Seppey et al. 2019) using the dataset for *Ascomycota* (ascomycota_odb10). Ab initio gene modeling was performed with Augustus v3.2.3 (Hoff and Stanke 2019) using the training dataset from *Aspergillus nidulans*. Based on these gene models, secondary metabolites such as type I and type III polyketide synthetases (PKSs), non-ribosomal peptide synthetases (NRPSs), terpene clusters, indole clusters, and fungal-RiPP peptides were predicted using antiSMASH v5.1.2 (Blin et al. 2019). All other gene models of the Augustus prediction were annotated with DIAMOND (Buchfink et al. 2014) searches against public databases, e.g. Swissprot, trEMBL, PFAM, TIGR, HAMAP, and CDD, and subsequent manual curation of each annotation. For comparative purposes, we also constructed a de novo assembly of the trimmed short-read Illumina sequences with SPAdes v3.11.1 (Nurk et al. 2013).

Genome identity was determined with phylogenetic analyses of the internal transcribed spacer (ITS) barcoding marker sequence (Schoch et al. 2012). The ITS region was identified with a BLASTn search using the *P. stellaris* ITS sequence (AY498689) as query. The complete ITS region (ITS1, 5.8S, and ITS2) was extracted from the *P. stellaris* assembly and aligned with the ITS sequences of 31 other *Physcia* species downloaded from NCBI (https://www.ncbi.nlm.nih.gov/). Sequences were aligned using MAFFT v7 (Katoh and Standley 2013) and Gblocks v0.91b (Talavera and Castresana 2007) was used to delimit and remove ambiguous nucleotide positions from the alignment. Maximum Likelihood analysis was conducted using RAxML v8.1.11 (Stamatakis 2014) on the CIPRES Science Gateway server (http://www.phylo.org/portal2). Nodal support was evaluated with 1000 bootstrap pseudoreplicates. The resulting phylogenetic tree was drawn with the program FigTree v1.4.2 (http://tree.bio.ed.ac.uk/software/figtree).

#### Results and discussion

**Fig. 6:**
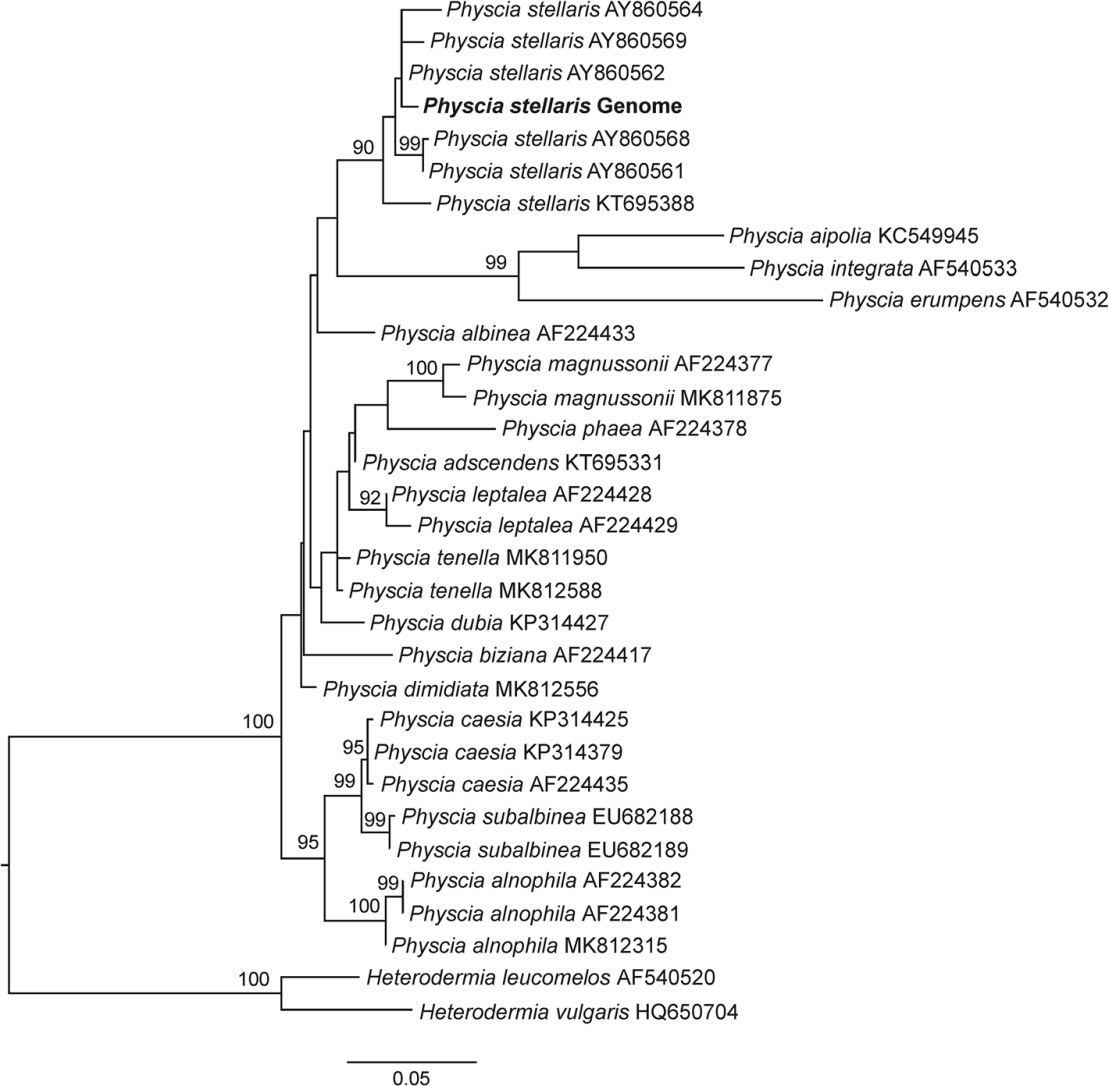
Maximum Likelihood tree based on ITS sequences of *Physcia* species including the genome sequence of *Physcia stellaris* reported here (shown in **bold**). Bootstrap values above 75% are indicated at the nodes. Two species of *Heterodermia* were used as an outgroup

We sequenced and assembled the first draft genome of the nitrogen-tolerant lichen fungus *Physcia stellaris*. The phylogenetic interference based on the extracted ITS region confirmed the sequenced draft genome as the lichenized fungal genome of *P. stellaris* (Fig. 6). The ITS of the sequenced genome clustered together with six other *P. stellaris* taxa with a bootstrap support of 90%. This clade of *P. stellaris* was clearly separated from a well-supported sister clade that contained *P. aipolia*, *P. integrata*, and *P. erumpens*.

The genome of the lichenized fungal culture #10–5 of *P. stellaris* assembled into 184 scaffolds with a total length of 46.57 Mb (Table 1). Scaffolding based on the long-read Nanopore data connected 12 of 196 contigs into scaffolds. The final assembly contained 94 scaffolds larger than 100 Kb, 38 scaffolds larger than 500 Kb, and 6 scaffolds larger than 1 Mb. The assembly had an N50 of 594.86 Kb and a GC content of 43.32%. The mean assembly coverage was 31.16x (SD = 62.35x) of Nanopore sequences and 30.25x (SD = 37.36x) of Illumina sequences. The variations in coverage of both mappings were due to smaller contigs that received lower coverage than average and the contig of the mitogenome that received much higher coverage than average. BUSCO analysis estimated a genome completeness of 95.6% by identifying 1617 complete and 3 fragmented genes from a total of 1706 *Ascomycota* BUSCO genes searched. Augustus predicted 10,522 genes in the assembly, resulting in an average gene density of 225.94 genes per 1 Mb. These genes were interrupted by 24,221 introns; hence each gene contained on average 2.3 introns.

**Table 1:** General features of the genome of *Physcia stellaris*

Assembly statistics	***Physcia stellaris*** 10–5
Assembly size (Mb)	46.57
Scaffolds	184
Contigs	196
Scaffold N50 (Kb)	594.86
Contig N50 (Kb)	586.07
Mean Coverage by Nanopore (fold)	31.16
Mean Coverage by Illumina (fold)	30.25
BUSCO coverage (%)	95.6
G + C content (%)	43.32
**Gene prediciton statistics**	
Predicted gene models	10,522
Average gene length (bp)	1760.35
Gene density (genes per Mb)	225.94
Predicted introns	24,441
Introns per gene	2.32
**Secondary metabolites (SM) statistics**	
Total SM clusters	74
Type I polyketide synthetases (PKSs)	20
Type III PKSs	1
Nonribosomal peptide synthetases (NRPSs)	15
NRPS-like fragments	16
Terpene clusters	17
Indole clusters	4
fungal-RiPP	1

The presented draft genome was sequenced and assembled from a combination of long-read Nanopore and short-read Illumina data. This hybrid assembly is highly improved compared to a short-read only assembly of *P. stellaris* that resulted in 24,595 scaffolds and a N50 of 8492 Kb. However, other short-read draft assemblies of lichen-forming fungi resulted in larger scaffolds indicating a more complete genome structure, for example *Pseudevernia furfuracea* with 46 scaffolds and a N50 of 1178.8 Kb (Meiser et al. 2017). *Pseudevernia furfuracea* was sequenced with higher coverage (~350x vs ~ 30x of *Physcia stellaris*). But higher sequencing coverage does not always improve genome assemblies: a second genome in the same study (*Evernia prunastri*) was sequenced with higher coverage of ~410x and resulted in a more scattered draft genome of 277 scaffolds with a N50 of 264.45 Kb (Meiser et al. 2017). The likely reason that some genomes assemble better than others is due to genome complexity (i.e. repetitive structures). The use of long-read sequencing improved the assembly of *P. stellaris* and solved some repetitive regions. However, other scaffolding methods such as Hi-C, linked-read technologies, or optical mapping may be required to further improve the assembly of *P. stellaris* and other repetitive fungal genomes and unravel the genomic complexity of these organisms (Thomma et al. 2016).

The genome contained 74 regions that had genes associated with secondary metabolite biosynthesis. These regions included 20 type I and a single type III polyketide synthetases (PKSs), 15 non-ribosomal peptide synthetases (NRPSs), and 16 NRPS-like fragments. In addition to these synthetases, antiSMASH identified one fungal-RiPP peptide and 17 terpene, and four Indole clusters. Previous Illumina-sequenced lichenized fungal genomes had yields from 11 PKS genes in *Peltigera membranacea* to 32 type I PKS and 2 type III PKS genes in the genome of *Cladonia uncialis* (Bertrand and Sorensen 2018).

The availability of the *P. stellaris* draft genome from this study will allow comparative genomic studies within *Physciaceae* and will add to the genomic database of lichenized fungi for future research in evolutionary biology. Moreover, this genome will facilitate future studies in understanding the genetic mechanisms behind the adaptation to high nitrogen environments.

*Authors*: Felix Grewe^*^, Isabel Distefano, Sabine Huhndorf, Yukun Sun, **Claudio Ametrano, Pradeep K. Divakar, and H. Thorsten Lumbsch**

*Contact: fgrewe@fieldmuseum.org

## IMA GENOME-F 13E

### Draft genome sequences for Australian and South American strains of *Teratosphaeria pseudoeucalypti*

#### Introduction

*Teratosphaeria pseudoeucalypti* is a dothideomycete pathogen that was first encountered in south and central Queensland, north-western Australia (Andjic et al. 2010a). It is now known to be distributed across Queensland, parts of New South Wales, and in at least three South American countries (Burgess and Wingfield 2017; Andjic et al. 2019). The severe leaf-blight and defoliation of *Eucalyptus* trees caused by *T. pseudoeucalypti* is very similar to symptoms associated with *T. destructans*, one of the most aggressive of all *Eucalyptus* leaf pathogens (Greyling et al. 2016; Andjic et al. 2019). Leaf-spots caused by *T. pseudoeucalypti* have red-purple margins, similar to those caused by *T. destructans*, and range from large, confluent leaf lesions to individual necrotic leaf spots, depending on the host species and the leaf age (Andjic et al. 2010a; Ramos and Perez 2015).

Phylogenetically, *T. pseudoeucalypti* resides within an economically important clade of *Teratosphaeria* leaf pathogens that also includes *T. destructans* and *T. nubilosa* (Aylward et al. 2019). It is sister to *T. eucalypti* and was initially mistaken for that species because of their similar spore morphologies (Andjic et al. 2010a). Based on PCR amplification of mating type genes, most species in this clade are believed to be heterothallic (Havenga et al. 2020), with the notable exception of *T. nubilosa* (Pérez et al. 2010).

Subsequent to its description in 2010, *T. pseudoeucalypti* emerged in the neighbouring regions of Brazil (Cândido et al. 2014), Uruguay (Soria et al. 2014), and Argentina (Ramos and Perez 2015) in rapid succession. Although it was known in tropical and subtropical regions of eastern Australia, the South American hosts included temperate species such as *E. globulus* (Cerasoli et al. 2016). It is, therefore, of concern that *T. pseudoeucalypti*, unlike *T. destructans*, presents a threat to cold-tolerant *Eucalyptus* clones.

*Teratosphaeria pseudoeucalypti* is an aggressive pathogen with the potential to infect numerous *Eucalyptus* species across different climatic zones. Knowledge of its life-cycle and genetic diversity is required to manage the current disease outbreaks and to prepare for future disease problems. The aim of this study was to generate whole genome sequences, for both mating types of *T. pseudoeucalypti*, that will serve as a resource from which to design molecular markers and to conduct whole genome comparisons.

#### Sequenced strains

**Uruguay**: Colonia, isolated from spots on *Eucalyptus camaldulensis* leaves, Apr. 2015, *C. Pérez* (CMW 49159 = UY2039 - culture).

**Uruguay**: Durazno, isolated from spots on the leaves of a *Eucalyptus* hybrid, May 2015, *C. Pérez* (CMW 49161 = UY2151 - culture).

**Australia**: *Queensland*: Miriam Vale: isolated from spots on the leaves of *Eucalyptus grandis* × *E. camaldulensis*, 2005, *G. Pegg* (CMW 51515 = MUCC610 - culture).

#### Nucleotide accession number

The genomic sequences of these *T. pseudoeucalypti* isolates have been deposited at DDJ/EMBL/GenBank under the accessions JABASB000000000, JABBMY000000000 and JABBMZ000000000. This paper describes the first versions of these genomes.

#### Material and methods

Fungal cultures are maintained in the collections of the Forestry and Agricultural Biotechnology Institute (CMW), University of Pretoria, South Africa, the EEMAC laboratory (UY), Facultad de Agronomia, Universidad de la Republica, Uruguay and the Murdoch University culture collection (MUCC), Australia. Cultures were grown on malt extract agar (Merck, Wadeville, South Africa) at 25 °C in the dark. After approximately 2 wk., mycelial mats were transferred to 2 ml Eppendorf tubes and freeze dried. DNA was extracted following the protocol used for *T. destructans* (Wingfield et al. 2018b). A NanoDrop ND-1000 spectrophotometer (ThermoFisher Scientific, Wilmington, VA) and Qubit® 2.0 Fluorometer (Invitrogen, Carlsbad, CA) were used to estimate the quality and quantity (respectively) of the extracted DNA.

All three *T. pseudoeucalypti* isolates were sequenced using the Illumina HiSeq 2500 platform at Macrogen (Seoul, Korea). The paired-end Illumina libraries for the two Uruguayan isolates (CMW49159 and CMW49161) had insert sizes of 350 bp and the target read length was 100 bp. Genome assembly was performed with SPAdes v3.12.0, applying k-values of 21, 33 and 55. Low coverage sequencing for the Australian isolate (CMW51515) was conducted using a 550 bp insert library and a read length of 250 bp. This isolate was assembled with SPAdes v3.14.0 and k-values 21, 33, 55, 77, 99 and 127. For all three isolates, read correction was performed with Trimmomatic v0.39 (Bolger et al. 2014) and the MismatchCorrector option was turned on during the SPAdes assembly. Genome coverage was calculated by aligning the raw reads to the assembled genomes with Bowtie v1.2.2 (Langmead et al. 2009) and determining depth with Mosdepth v0.2.7 (Pedersen and Quinlan 2018), while genome completeness was estimated with BUSCO v3.0.2 (Waterhouse et al. 2017) using the *Fungi*, *Ascomycota,* and *Capnodiales* datasets.

Genome annotation procedures, including repeat-finding, followed those described by Wingfield et al. (2019) for *T. gauchensis* and *T. zuluensis*. The putative mating type (*MAT1*) locus was identified by performing a BLASTn search against the genomes with the *MAT1–1* and *MAT1–2* idiomorphs of *T. destructans* as query sequences (GenBank accessions MN531144 and MN531145; Havenga et al. 2020). For phylogenetic analysis, we used the beta-tubulin, translation elongation factor-1α (EF-1α) and internal transcribed spacer (ITS) gene regions. Alignment and curation was performed on the NGPhylogeny.fr platform (Lemoine et al. 2019) using MAFFT 7.407_1 (Katoh and Standley 2013) and Gblocks 0.91.1 (Talavera and Castresana 2007). Alignments were concatenated with FASconCAT-G (Kück and Longo 2014), the best-fit nucleotide substitution model for each partition was determined with ModelTest-NG (Darriba et al. 2020) and a maximum likelihood phylogeny was calculated with RAxML-NG 0.9.0 (Kozlov et al. 2019).

#### Results and discussion

**Fig. 7:**
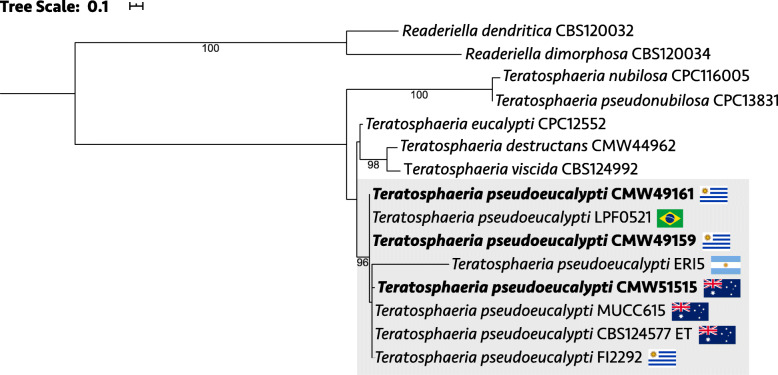
Maximum likelihood phylogeny of *Teratosphaeria pseudoeucalypti* and other closely related foliar pathogens. Isolates collected from the four countries in which *T. pseudoeucalypti* is known were included. Apart from the genomes generated in this study (shown in **bold**), the gene sequences were sourced from previous studies (Andjic et al. 2010a; Quaedvlieg et al. 2014; Soria et al. 2014; Ramos and Perez 2015; Aylward et al. 2019). ET = ex-type strain

In the phylogeny based on concatenated beta-tubulin, EF-1α and ITS sequences (Fig. 7), the three isolates selected for genome sequencing formed a well-supported (96%) clade with *T. pseudoeucalypti* isolates from across the distribution range of this species. Taxonomic relationships mirrored those previously described for species in this clade of economically important *Teratosphaeria* leaf pathogens (Quaedvlieg et al. 2014). The sister relationship between *T. pseudoeucalypti* and *T. eucalypti* was not resolved in this analysis, but has been supported in previous studies (Andjic et al. 2010b; Soria et al. 2014).

The genome assemblies of the *T. pseudoeucalypti* isolates from Uruguay were 26.8 Mb with >100x coverage, whereas the assembly of the Australian isolate totaled 30.2 Mb with approximately 22x coverage. The number of scaffolds, N50 and L50 was in the same range for all three genomes (Table 2). The difference in genome size is likely due to the different read lengths that were generated and used for genome assembly. Since the Australian isolate was sequenced with 250 bp reads, compared to 100 bp reads for the Uruguayan isolates, a greater number of repetitive regions would have been resolved in the genome assembly of the Australian isolate, hence its larger genome size.

**Table 2:** Assembly and annotation statistics^a^ for the *Teratosphaeria pseudoeucalypti* genomes

	CMW49159	CMW49161	CMW51515
**Isolate information**			
Origin	Uruguay	Uruguay	Australia
Mating type	*MAT1–1*	*MAT1–1*	*MAT1–2*
**Genome assembly**			
Assembly size (Mb)	26.78	26.79	30.24
Number of scaffolds	630	622	608
N50 (kb)	166.471	181.549	183.627
L50	44	43	52
Estimated coverage	×150	×120	×22
GC content	54.2%	54.2%	52.0%
**Genome annotation**			
Supported^b^ gene predictions	8879	8865	8870
Unsupported^c^ gene predictions	1356	1384	1569
Repetitive regions	2.3%	2.3%	12.25%
**BUSCO Genome completeness**			
*Fungi* odb10	98.94%	98.68%	98.68%
*Ascomycota* odb10	98.01%	98.18%	96.83%
*Capnodiales* odb10	93.21%	93.15%	94.46%

^a^All statistics are based on scaffolds > 499 bp

^b^Supported by external protein or EST evidence

^c^Predicted by de novo gene predictors, but without external evidence

An analysis of the repetitive regions in these genomes supported the conclusion that read length may be the cause of the difference in genome size observed. The Australian genome was 12.3% repetitive, whereas repetitive regions comprised only 2.3% of the other two genomes. A repeat content of 12.3% would be closer to the 17% estimated for the closely related leaf pathogen *T. destructans*, whereas lower repeat contents (ca. 2%) resemble those of the stem canker pathogens *T. gauchensis* and *T. zuluensis* (Wingfield et al. 2018b, 2019).

The three *T. pseudoeucalypti* genomes had a similar number of predicted genes, especially when considering the gene predictions that were similar to the proteins or CDS sequences of other species (~ 8870 predictions). A further 1356–1569 genes that did not have such external evidence were also predicted in the three species. Based on gene orthologs that should be present in certain taxa, the predicted completeness of the three genomes ranged from 93.2% at the highest taxonomic level (Capnodiales) to 98.9% at the lowest level (Fungi). The *MAT* idiomorphs in all three *T. pseudoeucalypti* isolates had > 96% nucleotide identity to the *T. destructans MAT1* idiomorphs and, therefore, resembled the *MAT1* loci of other heterothallic *Teratosphaeria* species (Aylward et al. 2020; Havenga et al. 2020). In the genomes of the two Uruguayan isolates, the *MAT1–1* idiomorph consisted of the *MAT1–1-1* and *MAT1–1-10* genes, whereas the *MAT1–2* idiomorph in the genome of the Australian isolate comprised the *MAT1–2-1* and *MAT1–2-12* genes.

*Teratosphaeria pseudoeucalypti* is the third *Teratosphaeria* foliar pathogen, after *T. destructans* (Wingfield et al. 2018b; Havenga et al. 2020) and *T. nubilosa* (Abdollahzadeh et al. 2020), for which whole genome sequence data are available. The *T. pseudoeucalypti* genomes generated in this study will be used to develop genetic markers to study the diversity of *T. pseudoeucalypti* outbreaks and to compare this pathogen with its closest relatives. Along with the genomes of *T. destructans*, *T. nubilosa* and the stem canker pathogens, *T. gauchensis* and *T. zuluensis* (Wingfield et al. 2019), the data generated in this study contribute to a growing database of knowledge concerning *Eucalyptus* disease-causing fungi.

*Authors:* Janneke Aylward*, **Tuan A. Duong**, **Brenda D. Wingfield**, **Nazaret Ramírez-Berrutti**, **Carlos A. Pérez**, and **Michael J. Wingfield**

*Contact: Janneke.Aylward@fabi.up.ac.za

## Data Availability

All data and materials are available in data banks and culture collections and fungaria, respectively. Relevant details are given in the contributions.
